# Assessment of requests for medication-related follow-up after hospital discharge, and the relation to unplanned hospital revisits, in older patients: a multicentre retrospective chart review

**DOI:** 10.1186/s12877-021-02564-5

**Published:** 2021-11-02

**Authors:** Henrik Cam, Thomas Gerardus Hendrik Kempen, Helena Eriksson, Kanar Abdulreda, Kristin Franzon, Ulrika Gillespie

**Affiliations:** 1 Hospital Pharmacy Department, Uppsala University Hospital, SE-751 85 Uppsala, Sweden; 2 Department of Pharmacy, Uppsala University, Uppsala, Sweden; 3 Academic Primary Health Care Centre, Region Uppsala, Uppsala, Sweden; 4 Geriatric Department, Uppsala University Hospital, Uppsala, Sweden; 5 Department of Public Health and Caring Sciences, Uppsala University, Uppsala, Sweden

**Keywords:** Aged, Aftercare, Continuity of patient care, Electronic health records, Hospitalisation, Patient discharge, Patient transfer, Pharmaceutical services, Patient safety, Referral and consultation

## Abstract

**Background:**

The discharge of older hospitalised patients is critical in terms of patient safety. Inadequate transfer of information about medications to the next healthcare provider is a known problem, but there is a lack of understanding of this problem in settings where shared electronic health records are used.

The aims of this study were to evaluate the prevalence of patients for whom hospitals sent adequate requests for medication-related follow-up at discharge, the proportion of patients with unplanned hospital revisits because of inadequate follow-up requests, and the association between medication reviews performed during hospitalisation and adequate or inadequate follow-up requests.

**Methods:**

We conducted a retrospective chart review. The study population was randomly selected from a cluster-randomised crossover trial which included patients 65 years or older who had been admitted to three hospitals in Sweden with shared electronic health records between hospital and primary care. Each patient was assessed with respect to the adequacy of the request for follow-up. For patients where the hospitals sent inadequate requests, data about any unplanned hospital revisits were collected, and we assessed whether the inadequate requests had contributed to the revisits. The association between medication reviews and adequate or inadequate requests was analysed with a Chi-square test.

**Results:**

A total of 699 patients were included. The patients’ mean age was 80 years; an average of 10 medications each were prescribed on hospital admission. The hospitals sent an adequate request for 418 (60%) patients. Thirty-eight patients (14%) had a hospital revisit within six months of discharge which was related to an inadequate request. The proportion of adequate or inadequate requests did not differ between patients who had received a medication review during hospitalisation and those who had not (*p* = 0.83).

**Conclusions:**

The prevalence of patients for whom the hospitals sent adequate follow-up requests on discharge was low. More than one in every ten who had an inadequate request revisited hospital within six months of discharge for reasons related to the request. Medication reviews conducted during hospitalisation did not affect the proportion of adequate or inadequate requests sent. A communication gap still exists despite the usage of a shared electronic health record between primary and secondary care levels.

## Background

Medications are the cause of up to 20% of all hospitalisations, including hospital revisits [1, 2]. Older patients have a high risk of adverse drug events (ADEs) primarily due to a high disease burden and consequent complex medication treatment [3]. The transition-of-care process, which is defined as a patient transferring to or returning from one healthcare setting to another, has been identified by the World Health Organization as a main area of focus for improving patient safety [4]. Specifically, the discharge of hospitalised patients has been identified as critical in terms of patient safety, mainly because of the increased risk of ADEs, and deficits in communication and information transfer between hospitals and primary care providers (PCPs) [4, 5]. Changes in patients’ medications are frequently made during hospitalisation [6]. This necessitates adequate follow-up planning and measures post-discharge, to prevent ADEs from occurring [7–10]. To increase patient safety, it is also of great importance to adequately involve and inform the patients about their medications and related decisions [11].

Discharge summaries are commonly used as a communication tool by the hospitals to communicate with the next healthcare provider and the patient [12, 13]. The discharge summary is transferred to the PCP and should include specific requests for active follow-up on the medication changes after discharge where necessary [14, 15]. The timely transfer of this discharge summary with clear requests for follow-up is considered crucial for PCPs to be able to process the information [16]. However, discharge summaries are commonly delayed, of deficient quality and contain incomplete information regarding follow-up plans [17, 18]. A shared electronic health record (EHR) between hospital and primary care has been proposed as a solution to these problems [19–21]. These shared EHRs, including shared medication lists, have been implemented in several regions in Sweden. Consequently, medical notes and discharge documents do not need to be transferred and can be accessed instantly by PCPs. However, in order for the PCPs to be notified and to actively follow up on the medication changes after patient discharge, the hospital physician (HP) is usually required to send an electronic referral (i.e. an MRR – medication-related referral), in addition to writing the discharge summary, to request that the PCP accepts the responsibility of performing the follow-up [22]. These requests for medication-related follow-up for specific patients are central to the hospitals’ follow-up planning. There are to our knowledge no previous studies evaluating these requests for medication-related follow-up sent at hospital discharge in a setting where a shared EHR is used. It is important to understand this aspect of medication-related information transfer at hospital discharge for the development of future interventions to improve the transition-of-care process.

One intervention which has been proposed to reduce medication-related morbidity is to undertake a comprehensive medication review (CMR) during hospitalisation [23–25]. The purpose is to optimise a patient’s medication treatment, to enable the best possible outcome and to minimise medication-related harm, by examining the pharmacotherapy in a structured and critical manner together with the patient [26]. Drug-related problems (DRPs), which potentially can result in ADEs, are identified and solved through the CMR process [27]. CMRs can result in more changes in the medication treatment and the identification of DRPs that should be resolved and/or followed-up in primary care than if a CMR is not performed. Consequently, it is of importance to have a thorough information transfer to the next healthcare provider. There is therefore a need to explore the impact of CMRs on information transfer at hospital discharge.

The primary aims of this study were to evaluate the prevalence of patients for whom hospitals sent adequate requests for medication-related follow-up at hospital discharge, regardless of the presence of a CMR during hospitalisation, and the proportion of patients who had an unplanned hospital revisit which could be related to an inadequate follow-up request. We also aimed to determine if there was an association between CMRs performed during hospitalisation and adequate or inadequate follow-up requests.

## Methods

### Study design and setting

This multicentre, retrospective study involved a review of the charts of patients admitted to Uppsala University Hospital, Enköping Hospital and Västerås Central Hospital in Sweden. Data collection and analyses were performed between September 2019 and September 2020.

Patients were selected from a multicentre, cluster-randomised, crossover trial (MedBridge) conducted between 2017 and 2018 at four Swedish hospitals: the hospitals in Uppsala, Enköping, Västerås and Gävle [28]. The aim of the trial was to study the effects of pharmacist-initiated hospital-based CMRs, including active follow-up, on older patients’ healthcare utilisation. Patients in the intervention groups had a pharmacist-initiated CMR, including a medication reconciliation before discharge, during hospitalisation and were compared with control patients without a CMR. In addition, the pharmacists had the possibility to send an MRR to the PCP at discharge in one of the intervention groups. The criteria for inclusion in the trial were patients aged 65 years or older and were admitted to one of the participating wards. A total number of 2637 patients were included. The incidence of unplanned hospital revisits within 12 months did not differ between the intervention groups and the control group [28]. A process evaluation of the trial [29] showed that in less than half of the intervention patients (46%), the CMR included a medication reconciliation at discharge. The pharmacist sent an MRR in only 6% of the patients in the intervention group that included this possibility. Furthermore, 15% of the control patients were contaminated, i.e. having received either a medication reconciliation or a CMR during hospitalisation.

The three hospitals included in this chart review are the hospitals of Uppsala and Enköping in Uppsala region and the hospital of Västerås in Västmanland region. These regions contain around 370,000 and 270,000 inhabitants, respectively [30]. The fourth hospital in the MedBridge trial, the hospital of Gävle, was excluded due to the use of an EHR system without the possibility to send electronic referrals to primary care. All three included hospitals use the same EHR system (Cambio Cosmic®, which is also used by most primary care centres in these regions). Requests for follow-up after hospital discharge are usually communicated by HPs to the next healthcare provider by sending electronic referrals through the EHR. The receiver/next healthcare provider then needs to respond to the referral by accepting or declining it. There is one exception to this system: sometimes an internal request (not sent via the EHR) is used when follow-up for the patient is planned with an outpatient specialist unit at the same hospital clinic from which the patient was discharged. When the MedBridge trial was initiated, the possibility for ward-based pharmacist to also send MRRs was introduced at the hospitals.

According to Swedish legislation, the discharge documents related to medications should consist of a discharge summary, a discharge letter with a medication report, and an updated medication list [12, 13]. These documents should be written in the patient’s EHR and sent to the next healthcare provider and the patient on the day of discharge. In both regions, these documents are available on the shared EHR, but the PCP is not actively notified when the discharge documents have been written.

### Study population and randomisation

**Fig. 1:**
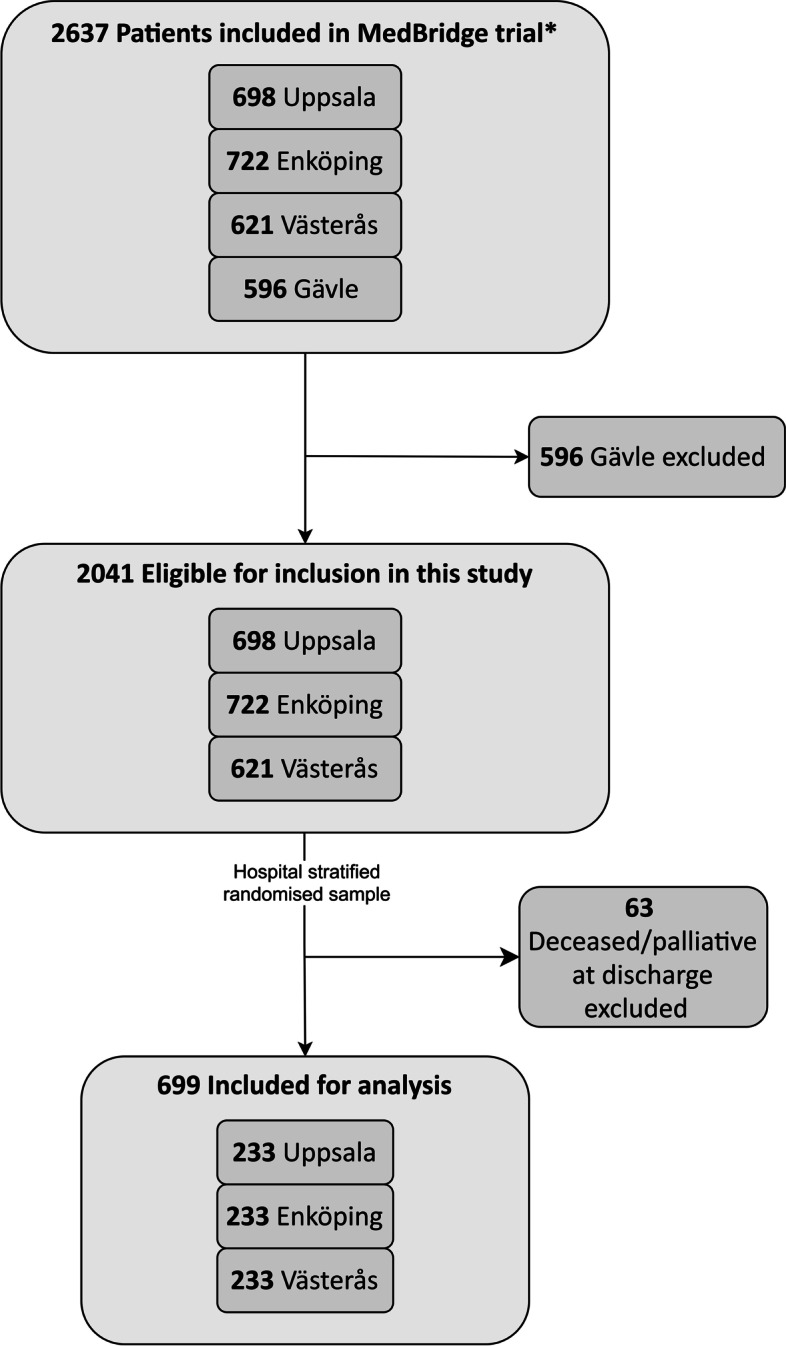
Flowchart of participants in the study. Values in each box are numbers of patients. * A multicentre, cluster-randomised, crossover trial [28]

The 2041 patients included in the MedBridge trial from the hospitals of Uppsala, Enköping and Västerås were eligible for inclusion in our study (Fig. 1). The eligible patients were stratified by hospital and randomly selected using Microsoft Excel® to generate random lists regardless of which study group the patient belonged to in the MedBridge trial. The following exclusion criteria were applied: deceased during hospitalisation or receiving palliative care on discharge. The inclusion process was stopped when one third of the eligible patients were included.

### Main outcomes

The main outcomes were proportion of patients with adequate and inadequate requests for follow-up and proportion of patients who had an unplanned hospital revisit within six months after hospital discharge which could be related to an inadequate follow-up request.

### Data collection

#### Detection of MRRs

Basic demographic data, hospital length of stay, medications at admission, previous medical history, data about which patients received a CMR during hospitalisation, and had an MRR sent to their PCP had already been collected for all participants in the MedBridge trial. This data-collection step had been performed by research assistants manually assessing the patients’ EHRs. An MRR sent to the PCP was defined as an electronic referral with a request for follow-up concerning the patient’s medications that was sent to the PCP at discharge. We also checked the records of all patients for whether an MRR had been sent via the EHR to any outpatient specialist unit at discharge. If the discharge summary mentioned that follow-up of the medications was planned for an outpatient specialist unit belonging to the same clinic as the ward from which the patient was discharged (without a referral in the EHR), this was classified as an MRR that had been sent.

#### Assessment of requests for follow-up

A comprehensive assessment of the adequacy of the request for follow-up was carried out for each patient. This was done by comparing the content of all the MRRs sent for each patient with the information available on the medication list and all written documentation by healthcare professionals in the EHR during hospitalisation. A request for follow-up was classified into four different categories: 1) adequate – complete MRRs sent 2) adequate – no MRRs sent, not needed 3) inadequate – incomplete MRRs sent and 4) inadequate – no MRRs sent, but needed. A request was deemed as “inadequate – incomplete MRRs sent” if the MRRs did not mention all the medication changes that needed follow-up or did not mention all relevant unresolved DRPs identified during hospitalisation, despite a statement in the EHR that the DRP would be communicated to the next healthcare provider. A medication change was defined as a medication being stopped or started or a change of dosage or formulation during hospitalisation, with the change lasting after discharge. A request was deemed as “inadequate – no MRRs sent, but needed” when no MRR had been sent and the assessment concluded that an MRR should have been sent to ensure adequate follow-up. The discharge summaries and discharge letters for the identified internal MRRs were also reviewed for follow-up requests and actions taken concerning medications.

#### Assessment of unplanned hospital revisits

Data were collected from the EHR about any unplanned hospital revisits (hospitalisations or emergency department visits) within six months of hospital discharge for patients with an inadequate request for follow-up. The EHR was also checked for follow-up actions taken by the PCP or outpatient specialist unit for these patients with an inadequate request. The reason for this was to confirm that no adequate actions had been taken despite the inadequate request. Based on this information an assessment was made whether the inadequate request (resulting in no or inadequate follow-up actions) was likely to have contributed to the unplanned revisit. Consequently, the revisits were classified as related or unrelated to the inadequate request. Only the first unplanned hospital revisit after hospital discharge could be classified as related to the inadequate request. The reason behind this was that additional changes in the medication treatment could have been made as a result of the revisit. Thus, each patient could only have one unplanned hospital revisit related to the inadequate request.

#### Data collectors and assessors

Data collection and assessments were made by either one senior clinical pharmacist (UG or HC) or one of two final-year pharmacy students (HE or KA) who were trained and supervised by HC. Any cases where a conclusion could not be drawn were discussed within an internal expert panel consisting of HC, UG and a physician specialising in geriatrics (KF). A decision was made when consensus was reached in each case.

### Statistical analysis

Data were analysed using descriptive statistics and are presented with a confidence interval of 95% where applicable. The association between CMRs performed during hospitalisation and inadequate or adequate requests for follow-up was analysed with a Chi-square test of independence (α = 0.05). All statistical analyses were made in Microsoft Excel.

### Ethics approval

All procedures involving data from human participants in this study were carried out in concordance with the ethical standards of Swedish legislation and the WMA declaration of Helsinki [31]. Informed written consent was obtained from all participants in the MedBridge trial. The trial was approved by the Swedish Central Ethical Review Board (CEPN; registration number: Ö21–2016).

## Results

Out of 2041 eligible patients, 762 were randomised for inclusion. Sixty-three patients (8%) were deceased or in a palliative stage at discharge and were excluded. This resulted in a total of 699 included patients. The baseline characteristics of the study population are shown in Table 1. The mean age of the study population was 80 (standard deviation, SD, ± 8) years, 50% (*n* = 218) were female, had an average of 10 (SD ± 6) prescribed medications, 68% (*n* = 476) had ≥3 diagnoses, the median duration of hospitalisation was 7 (interquartile range 4–12) days, and 437 (63%) had had a CMR during hospitalisation.

**Table 1:** Baseline characteristics of included patients

Characteristics	n (total = 699)	%
Age, mean years (SD)	80 (± 8)	N/A
Female sex	351	50
**Social support**		
Home care	179	26
Nursing home	81	12
Medications at admission, mean number (SD)	10 (±6)	N/A
Automated dose-dispensed medications	183	26
Hospitalisation duration, median days (IQR)	7 (4–11)	N/A
eGFR (ml/min) upon admission, mean (SD)	50 (25)	N/A
**CMR during hospitalisation**		
Yes	437	63
No	262	37
**Number of diagnoses in medical history**		
None	8	1
≥ 3	476	68
≥ 6	103	15
**Hospital**		
Uppsala	233	33
Enköping	233	33
Västerås	233	33

*Abbreviations*: *CMR* comprehensive medication review, *eGFR* estimated glomerular filtration rate, *IQR* interquartile range, *SD* standard deviation

Out of all the patients, 600 (86%) had at least one medication change during hospitalisation (Table 2). The mean number of medication changes was 3 (SD ± 3). An MRR was sent only to the PCP for 201 (29%) patients, only to a hospital outpatient unit for 189 (27%) patients, and to both the PCP and a hospital outpatient unit for 69 (10%) patients. For 240 (34%) patients, no MRR was sent. The hospitals had sent an adequate request for follow-up for 418 (60%) of the patients. Out of the 281 (40%) patients where the hospitals sent an inadequate request for follow-up, 167 (59%) had incomplete MRRs sent and 114 (41%) had no MRR sent, but needed one.

**Table 2:** Measures related to medication-related requests for follow-up

Measures	n (total = 699)	% (95% CI)
**Medication changes**		
Mean number (SD)	3 (± 3)	N/A
At least one medication change	600	86 (83–88)
**Sending of MRR**		
MRR sent only to PCP	201	29 (25–32)
MRR sent only to hospital outpatient unit	189	27 (24–30)
MRR sent to both PCP and hospital outpatient unit	69	10 (8–12)
No MRR sent	240	34 (31–38)
**Assessment of request for follow-up**		
Adequate request for follow-up	418	60 (56–63)
Complete MRRs sent	292	42 (36–48)
No MRR sent, not needed	126	18 (14–22)
Inadequate request for follow-up	281	40 (37–44)
Incomplete MRRs sent	167	24 (19–29)
No MRR sent, but needed	114	16 (13–20)
**Unplanned hospital revisits related to an inadequate request for follow-up** ^**a**^		
ED visits	9	1 (0.5–2)
Hospitalisation	29	4 (3–6)
Total unplanned hospital revisits	38	5 (4–7)

Values are number of patients

*Abbreviations*: *CI* confidence interval, *ED* emergency department, *MRR* medication-related referral, *PCP* primary care provider

^a^ Within six months of hospital discharge

Thirty-eight patients (5% of the study population or 14% of those with an inadequate request sent by the hospitals) had an unplanned hospital revisit within six months of hospital discharge which was related to an inadequate request. Examples of these cases are presented in Table 3.

**Table 3:** Examples of patients with an unplanned hospital revisit related to an inadequate request for follow-up

Patient	Hospitalisation course	Request for follow-up	Unplanned revisit
77 y, female Medical history: Heart failure, diabetes type 1, hypothyroidism, atrial fibrillation No. of medications at admission: 12	Admitted to hospital for syncope and diagnosed with a brain tumour. Betamethasone and levetiracetam were started as a direct result of the tumour. The insulin dosage was increased. Major changes were made in the heart failure treatment because of complications; spironolactone and enalapril were withdrawn because of hyperkalaemia, and the bisoprolol dosage was decreased because of bradycardia.	The Neurosurgery and Endocrinology departments were requested to follow up the medication changes associated with the brain tumour and diabetes, but an MRR for the heart failure medication changes was not sent.	Readmitted to hospital due to worsening of heart failure and was restarted on spironolactone; candesartan and furosemide were prescribed, and the dosage of bisoprolol was increased.
84 y, male Medical history: Previous myocardial infarction, renal failure, heart failure, hypertension, gout No. of medications at admission: 16	Admitted to hospital with a myocardial infarction. Worsening of heart and renal failure were detected. Treatment with candesartan and other new medications for the myocardial infarction was initiated. The patient’s gout treatment was also optimised by changing prednisolone to low dose allopurinol 100 mg once daily.	The heart failure outpatient unit was requested to follow up the new cardiology medications, but an MRR for the changed gout treatment was not sent.	Readmitted to hospital with joint pain due to worsening of gout.
82 y, female Medical history: Recent cerebral infarction with a subsequent hemiplegia and expressive aphasia No. of medications at admission: 6	Admitted from a short-term nursing facility after post-stroke seizures. An anticonvulsant was started. The patient was expressing pain and anxiety. The fentanyl dosage was increased, oxycodone as needed and oxazepam were started. Laxative treatment with macrogol as needed was unchanged.	No follow-up of the medications was requested. The patient returned to the short-term nursing facility after discharge.	Revisited the emergency department with abdominal pain due to constipation, possibly related to the opioid treatment.
91 y, male Medical history: Asthma, COPD, hypertension, dementia No. of medications at admission: 11	Admitted with COPD exacerbation. Poor inhaler technique was identified, and the inhalers were switched from DPIs (budesonide/formoterol) to pMDIs (budesonide/formoterol). The patient had problems with swollen ankles, which was suspected to be related to felodipine. An MRR to the PCP to consider change of antihypertensive medication was planned.	No MRR was sent to follow up the new inhalers and the swollen ankles.	Readmitted with a new COPD exacerbation and worsening of the swollen ankles.

*Abbreviations*: *COPD* chronic obstructive pulmonary disease, *DPI* dry-powder inhaler, *MRR* medication-related referral, *pMDI* pressurised metered-dose inhaler, *PCP* primacy care provider

As shown in Table 4, CMR performed during hospitalisation did not affect the proportion of patients with an adequate or inadequate follow-up request (*p* = 0.83).

**Table 4:** Association between adequate/inadequate follow-up requests and patients with or without CMR

Adequacy of follow-up requests	With CMR	Without CMR	Chi^**2**^ test of independence
Adequate request	260 (37, 34–41)	158 (23, 20–26)	χ^2^ (1, *n* = 699) = .04^a^ *p* = .83
Inadequate request	177 (25, 22–29)	104 (15, 12–18)	

Values are number of patients (%, 95 CI).

*Abbreviations*: *CI* Confidence Interval, *CMR* Comprehensive Medication Review

^a^ α = .05

## Discussion

This study evaluated the prevalence of patients for whom hospitals sent adequate requests for medication-related follow-up at hospital discharge, regardless of the presence of a CMR during hospitalisation, the proportion of patients who had an unplanned hospital revisit which could be related to an inadequate follow-up request, and the presence of an association between CMRs carried out during hospitalisation and adequate or inadequate requests. We found that requests for follow-up were adequate in 60% of the patients. Fourteen percent of the patients for whom the hospitals had sent an inadequate request had an unplanned hospital revisit within six months of discharge which could be related to the inadequacy.

The proportion of patients for whom the hospitals sent inadequate requests appears to be substantial and implies poor information transfer at hospital discharge. This is in agreement with previous findings from other countries where up to 50% of discharge summaries lacked follow-up planning for medications after hospital discharge [5, 17, 20, 32]. Also, for 34% of the patients, no MRRs were sent at all, despite an MRR was deemed necessary for about half of these patients (114/240) to ensure the required follow-up. Medication changes were common, as expected considering the older, multimorbid patient population. Nine out of ten patients had at least one change in their medication treatment and the mean number of changes per patient (3) was well in line with previous studies of the same age group [6, 33, 34]. The high prevalence of medication changes during hospitalisation indicates the importance of a thorough and well structured follow-up process after the discharge of older patients. Hospitals should take into consideration that changes in patients’ medications often require follow-up to ensure that the changes do not result in adverse outcomes. There could be several reasons for not sending an MRR despite having made changes. The HPs and pharmacists may have intended to send an MRR but forgot, have assessed that follow-up on the changes by the PCP was unnecessary, or (in agreement with the patients or caregivers) have encouraged the patients to approach the PCP themselves after discharge. The discontinuous workflow of hospital physicians caring for patients during hospitalisation could also have contributed to this high proportion of inadequate requests. Discharge summaries and MRRs are sometimes written by physicians who have not been involved in the care of the patients until the day of discharge. The discharge documents are also frequently written by junior physicians with the least experience and with little formal training in writing them [35, 36].

Sending MRRs to the PCP or to other healthcare providers, in addition to the more commonly used discharge documents, is likely to be unique to the Swedish healthcare system. This additional administrative step for HPs does not seem to sufficiently enhance the quality of the communication about medications at hospital discharge. Yet PCPs stress the need for being notified after patient hospitalisation to ensure that a follow-up is initiated [19]. The notification does not happen automatically, not even if the patient’s medications were changed substantially [19, 37]. It is unclear if all HPs are aware of this fact. In our study, a shared EHR was used, but a notification in the form of a referral is still requested by the PCP. The high frequency of incomplete MRRs showed that a shared EHR does not automatically solve this problematic communication gap between hospital and PCP. The reason may be that there are no explicit discharge guidelines on when to send an MRR to the PCP after patient hospitalisation, or on what should be included in the MRR. PCPs have highlighted the importance of clear and structured documentation regarding which medications need to be followed up after discharge, and the recommended timing for follow-up [16]. They have also requested information on all the changes made and the reasons for them. The Royal College of Physicians in the UK has developed guidelines for the structure and content of patient records, including how a request for follow-up after discharge should be written, to mitigate any communication failures [38]. Development and subsequent implementation of equivalent standard guidelines on when and how to write MRRs could enhance their use and quality in a Swedish context.

The high proportion of patients for whom the hospitals sent inadequate requests is worrying since it is then up to the patient or the patient’s caregiver for initiating a follow-up of medication changes. It presupposes that the patient or caregiver fully understand what the changes were, why they were carried out, and that follow-up is needed after hospital discharge. For this to happen successfully, the information transfer to the patient or caregiver needs to be clear and correct, which has been shown persistently is not the case [39–41]. The quality of written discharge information to patients is often poor [39, 41], and discharge consultations are often performed without clear and sufficient instructions concerning follow-up [42, 43] and are often presented abruptly without preparing the patients for self-management of their medications [44]. In addition, one could argue that the patient or caregiver always has the responsibility for medication treatment and should ask for follow-up if they are not contacted by their PCP after discharge. However, it has been shown that older patients often assume that hospital physicians are obligated to communicate with their PCP and that this communication will be smoothly carried out [45]. It has also been shown that older patients rarely question HPs and assume that they always make the correct decisions [46]. It may therefore be unhelpful to assume that this patient group will initiate contact with the PCP for follow-up of medication changes, unless an obvious side effect occurs. The lack of clear and explicit information about the necessity of follow-up can result in a significant risk of medication-related morbidity.

We found that 5% of the study population or more than one tenth of the patients for whom the hospitals sent an inadequate request returned to the hospital within six months for reasons that was likely to be connected to the inadequate request. This finding is substantial, and one can see the potential of reducing hospital revisits by improving the adequacy of the requests. Previous studies of the effects of discharge documents on hospital revisits have been contradictory. Several earlier studies have not shown an association between poor transfer of discharge documentation and hospital revisits [47–49], but these were conducted during a time and a setting when patients were cared for by their PCP during hospitalisation. A more recent study by Salim et al. showed that better quality discharge documentation could prevent one third of rehospitalisations in patients with heart failure exacerbations [50]. In that study, as in ours, patients were cared for by HPs.

This study is, to our knowledge, the first to investigate an association between CMRs carried out during hospitalisation and inadequate requests sent for follow-up. The fact that no positive link was found indicates that CMRs were not responsible for loss of follow-up after discharge. On the other hand, CMRs did not improve the chances of an adequate request being sent either. The CMRs carried out during hospitalisation in the MedBridge trial were supposed to include a medication reconciliation on hospital discharge. This should have involved a review of all the patient’s medications and a check that there was a plan to follow up on all the relevant medication changes made during hospitalisation. However, process evaluation of the MedBridge trial showed that only 50% of the patients who had a CMR actually received a medication reconciliation at discharge, and only 6% of the MRRs to PCP were sent by pharmacists performing the CMR [29]. It is important to stress the need to focus on the discharge process when carrying out a CMR during hospitalisation [25], since substantial medication changes may be made as a result. To improve patient safety by improving communications about medication changes to the next healthcare provider, it is not enough to carry out a CMR as in the MedBridge trial. A more focused intervention on the discharge process is needed.

### Limitations

There are some limitations of this study. Firstly, there are limitations associated with retrospective chart reviews. The results were based on what the healthcare professionals wrote in their notes, referrals, and other sections of the EHR - and on our interpretation of these. There may have been valid reasons not to send an MRR to the next healthcare provider which we could not possibly know if they were not documented. We were not able to take into account what was only communicated verbally to a patient. Moreover, there were four researchers involved in the collection of data, and any inconsistencies in assessment may have affected the data quality. We tried to mitigate this by training those involved in data collection and by discussing any unclear cases in an internal expert panel until consensus was reached.

Secondly, we did not investigate how the adequate requests for follow-up were handled by the next healthcare provider: whether the requests were accepted or rejected, or whether and how the follow-up was carried out. An adequate request for follow-up does not automatically mean that the transfer of discharge documentation was unproblematic. The next healthcare provider needs to process and act on the request for follow-up in a correct and timely manner. It has previously been shown that PCPs fail to complete tests and follow-up appointments that the hospital has requested for one quarter of discharged patients [15].

Thirdly, this study design only showed a possible connection between unplanned hospital revisits and patients for whom the hospitals sent inadequate requests for follow-up. An unplanned hospital revisit may have happened even if the request was adequate or if a follow-up was perfectly carried out. We did not investigate the number of unplanned hospital revisits among patients for whom the hospitals sent adequate requests and, therefore, could not make this comparison.

### Implications for research and practice

This study adds to the evidence of deficiencies in the communication between hospitals and the next healthcare provider on hospital discharge. A problematic communication gap still exists, despite the usage of a shared EHR system between hospital care and primary care. Future research should focus on finding ways to improve the adequacy of requests for follow-up and discharge documentation overall, and to subsequently include these in clinical practice. This study also addresses the need for the development and implementation of guidelines on when and how to write MRRs for hospital discharge.

## Conclusions

In this study of older hospitalised patients, the prevalence of patients for whom the hospitals sent adequate requests for follow-up on discharge was low, and more than one out of ten inadequate requests was associated with an unplanned hospital revisit within six months of discharge. CMRs conducted during hospitalisation did not affect the proportion of patients for whom the hospitals sent inadequate or adequate requests. A problematic communication gap still exists, despite the usage of a shared EHR system between primary and secondary care levels.

## Data Availability

The datasets generated and/or analysed during this study are not publicly available since sharing of data was not included in the approval from the ethics committee. However, they are available from the corresponding author on reasonable request. All authors received administrative permission to access and use all the data in this study.

## Abbreviations

ADE: Adverse Drug Event
COPD: Chronic obstructive pulmonary disease
CMR: Comprehensive medication review
DRP: Drug-related problem
EHR: Electronic health record
HP: Hospital physician
MRR: Medication-related referral
PCP: Primary care provider
SD: Standard deviation
